# KRT6A Restricts Influenza A Virus Replication by Inhibiting the Nuclear Import and Assembly of Viral Ribonucleoprotein Complex

**DOI:** 10.3390/v17050671

**Published:** 2025-05-04

**Authors:** Yu Chang, Zhibo Shan, Wenjun Shi, Qibing Li, Yihan Wang, Bo Wang, Guangwen Wang, Hualan Chen, Li Jiang, Chengjun Li

**Affiliations:** 1 State Key Laboratory for Animal Disease Control and Prevention, Harbin Veterinary Research Institute, Chinese Academy of Agricultural Sciences, Harbin 150069, China; changyu204@sina.com (Y.C.); szb1225@163.com (Z.S.); qlibevis@163.com (Q.L.); wangyihan0825@163.com (Y.W.); wangbocaas@163.com (B.W.); wangguangwen@caas.cn (G.W.); chenhualan@caas.cn (H.C.); 2 Technology Center of Qingdao Customs, Qingdao 266000, China; duoyanji@126.com

**Keywords:** influenza A virus, KRT6A, vRNP complex, NP, nuclear import, assembly

## Abstract

The transcription and replication of the genome of influenza A virus (IAV) take place in the nucleus of infected cells, which is catalyzed by the viral ribonucleoprotein (vRNP) complex. The nuclear import of the vRNP complex and its component proteins is essential for the efficient replication of IAV and is therefore prone to be targeted by host restriction factors. Herein, we found that host cellular protein keratin 6A (KRT6A) is a negative regulator of IAV replication because siRNA-mediated knockdown of *KRT6A* expression increased the growth titers of IAV, whereas exogenous overexpression of KRT6A reduced viral yields. The nuclear import of incoming vRNP complexes and newly synthesized nucleoprotein (NP) was significantly impaired when KRT6A was overexpressed. Further studies showed that KRT6A interacts with the four vRNP complex proteins—polymerase basic protein 1 (PB1), polymerase basic protein 2 (PB2), polymerase acidic protein (PA), and NP. Notably, the interaction between KRT6A and vRNP complex proteins had no effect on the nuclear import of PB2 or the PB1-PA heterodimer but impaired the interaction between NP and the nuclear import adaptor importin α3, thereby inhibiting the nuclear import of incoming vRNP complexes and newly synthesized NP. Moreover, KRT6A was further shown to suppress the assembly of the vRNP complex and consequently reduce viral polymerase activity. Together, our data uncover a novel role of KRT6A in counteracting the nuclear import and functions of the vRNP complex, thereby restricting the replication of IAV.

## 1. Introduction

Influenza A virus (IAV) is an important zoonotic pathogen that belongs to the family Orthomyxoviridae. The genome of IAV consists of eight single-stranded negative-sense RNA segments, encoding ten essential proteins and up to eight accessory proteins [1]. Based on the antigenicity of the two surface glycoproteins, hemagglutinin (HA) and neuraminidase (NA), IAV is classified into different subtypes. So far, 19 HA subtypes and 11 NA subtypes of IAV have been identified [2,3]. Since 1918, only H1N1, H2N2, H3N2, and H1N2 subtypes of IAV have circulated in humans [4], causing seasonal epidemics and/or occasional pandemics. However, the widespread global circulation of avian influenza viruses in recent years has led to the appearance of more and more subtypes of IAV (i.e., H5N1, H5N8, H7N9, H9N2, H3N8, and H10N8) capable of infecting or even killing humans [5,6,7,8,9,10,11,12,13].

The three viral polymerases [polymerase basic protein 1 (PB1), polymerase basic protein 2 (PB2), and polymerase acidic protein (PA)], along with nucleoprotein (NP) and viral RNA, together constitute the viral ribonucleoprotein (vRNP) complex, which is responsible for the transcription and replication of the viral genome. In the replication cycle of IAV, upon the completion of the uncoating step, the incoming vRNP complex is released into the cytoplasm and subsequently delivered into the nucleus through the classical nuclear import pathway [14,15]. The translocation of the vRNP complex into the nucleus is mediated by viral NP protein, which harbors two nuclear localization signals (NLSs); one is an unconventional NLS located between amino acids 3–13 at the N terminus [16,17], and the other is a bipartite NLS that sits between amino acids 198 and 216 [18]. The NLSs of NP bind to isoforms of importin α, which further recruits importin β, leading to the ultimate transport of the incoming vRNP complex or NP itself into the nucleus through the nuclear pore complex [14,15].

The vRNP complex lies in the center of the IAV replication cycle. Within the vRNP complex, NP is the most abundant component protein and serves as the carrier of viral RNA [19]. Several amino acid mutations in NP (e.g., A286V, T437M, and Q357K [20,21]) have been identified to be important for the pathogenicity of IAV. To efficiently replicate in the host, IAV uses NP to interact with proviral host factors. For example, the association between NP and Bcl10-interacting protein with CARD 1 (BinCARD1) enhances the binding between NP and importin α7, thereby facilitating the nuclear import of the vRNP complex and newly synthesized NP [22]; Tat stimulatory factor 1 (Tat-SF1) interacts with free NP but not NP bound to RNA, and acts as a molecular chaperone to promote the formation of NP–RNA complexes [23]; DEAD-box RNA helicase U2AF65-associated protein (UAP56) associates with NP and enhances viral RNA synthesis [24]; high-mobility group box 1 protein (HMGB1) interacts with NP in the nucleus to promote the recruitment of the vRNP complex at chromatin transcriptional active sites, thus enhancing viral polymerase activity and growth [25]; and fragile X mental retardation protein (FMRP) binds NP to promote the assembly and nuclear export of the vRNP complex [26]. On the other hand, the crucial role of NP in the replication cycle of IAV also makes it an important target of the host restriction factors in combating IAV infection. Among them, Moloney Leukemia virus 10 (MOV10) and phospholipid scramblase 1 (PLSCR1) interact with NP and interfere with its binding with importin α or the further recruitment of importin β, thereby suppressing the nuclear import of the NP/vRNP complex and inhibiting the replication of IAV [27,28]; tripartite motif-containing 4 (TRIM4), TRIM14, TRIM22, and TRIM41 interact with and mediate polyubiquitination of NP, leading to its proteasomal degradation [29,30,31,32].

Keratin proteins are the predominant subgroup of intermediate filaments (IFs) [33], and consist of two types: type I (28 members) and type II (26 members) [34]. All type I keratin genes, except the gene encoding KRT18, are clustered on the long arm of chromosome 17, whereas all type II keratin genes, together with the gene encoding KRT18, are located on the long arm of chromosome 12 [35]. KRT6A is a member of type II keratins [34]. It is reported that KRT6A modulates the migration of keratinocytes in response to injury [36]. The expression of KRT6A has been shown to be significantly upregulated in non-small cell lung cancer (NSCLC) and colorectal cancer [37,38], and its expression promotes cell proliferation and invasion in NSCLC and nasopharyngeal carcinoma [39,40]. Of importance, the high expression level of KRT6A in NSCLC is correlated with poor patient prognosis [37]. However, whether KRT6A plays a role in virus infection is still largely unknown.

In the present study, we demonstrated that KRT6A is a novel restriction factor for the replication of IAV. KRT6A interacted with the four vRNP complex proteins of IAV, and the interaction between KRT6A and NP specifically impaired the association between NP and importin α3, thereby inhibiting the nuclear import of the incoming vRNP complex and NP itself. Furthermore, the expression of KRT6A also hindered the assembly of the vRNP complex and consequently reduced the viral polymerase activity. Our study thus unveiled that KRT6A is a negative regulator of IAV replication by modulating the nuclear import and function of the vRNP complex.

## 2. Materials and Methods

### 2.1. Cells and Virus

A549, HEK293T, HeLa, and MDCK cells were cultured in F12K (Gibco, Grand Island, NY, USA) with 10% fetal bovine serum (FBS, Gibco) (A549), DMEM (Sigma-Aldrich, St. Louis, MO, USA) containing 10% FBS (HEK293T, HeLa), and DMEM containing 5% bovine calf serum (BCS, Sigma-Aldrich) (MDCK). All media were supplemented with 100 units/mL penicillin and 100 μg/mL streptomycin (Gibco). All cells were cultured at 37 °C with 5% CO2. A/WSN/1933 (WSN, H1N1) virus was propagated in MDCK cells, as previously described [41].

### 2.2. Plasmids and Small Interfering RNAs

The open reading frame (ORF) of *KRT6A* was inserted into the pCDNA3.1 vector with a Myc tag at the N-terminus. The ORFs of *PB2*, *PB1*, *PA*, and *NP* derived from the WSN (H1N1) virus were cloned into the mammalian expression vector pCAGGS as described previously [27]. Plasmids pCAGGS-V5-WSNPB1, pCAGGS-V5-WSNPA, pCAGGS-V5-WSNNP, pCAGGS-Flag-importin α1, pCAGGS-Flag-importin α3, pCAGGS-Flag-importin α5, and pCAGGS-Flag-importin α7 were generated by inserting the ORFs of *PB1*, *PA*, *NP* of WSN (H1N1) virus, *importin α1*, *importin α3*, *importin α5*, and *importin α7* fused with a V5 or Flag tag sequence at the N-terminus into the pCAGGS vector. Plasmids pCAGGS-WSNPA-Flag and pCAGGS-WSNNP-Flag were generated by inserting the ORF of *PA* and *NP* of WSN (H1N1) virus fused with a C-terminal Flag tag sequence into the pCAGGS vector. The generation of pHH21-SC09NS-F-Luc, used to produce virus-like negative-sense RNA harboring a firefly luciferase reporter gene, has been described previously [27]. All plasmid constructs were confirmed by sequencing. The small interfering RNA targeting *KRT6A* (si_*KRT6A*, sense: 5′-CCAGCAGGAAGAGCUAUATT-3′; antisense: 5′-UAUAGCUCUUCCUGCUGGTT-3′) and a scrambled siRNA (si_control, sense: 5′-UUCUCCGAACGUGUCACGUTT-3′; antisense: 5′-ACGUGACACGUUCGGAGAATT-3′) were purchased from GenePharma (Shanghai, China).

### 2.3. Antibodies

Mouse monoclonal antibodies (mAbs) and rabbit polyclonal antibodies (pAbs) against the following IAV proteins were generated in our laboratory: PB2, PB1, PA, and NP [42]. Mouse anti-V5 mAb (SAB2702199), mouse anti-Myc mAb (M4439), and rabbit anti-Flag pAb (F7425) were from Sigma Aldrich. Rabbit anti-Myc pAb (16286-1-AP), mouse anti-KRT6A mAb (66685-1-lg), rabbit anti-KRT6A pAb (10590-1-AP), rabbit anti-GAPDH pAb (10494-1-AP), mouse anti-GAPDH mAb (60004-1-Ig), and rabbit anti-LaminB1 pAb (12987-1-AP) were from Proteintech (Wuhan, China). Rabbit anti-V5 pAb (AB3792) and mouse anti-Flag mAb (B3111) were from Millipore (Darmstadt, Germany). Alexa Fluor 633 goat anti-mouse IgG (H + L) (A21050), Alexa Fluor 633 goat anti-rabbit IgG (H + L) (A21071), Alexa Fluor 488 goat anti-rabbit IgG (H + L) (A11034), and Alexa Fluor 488 goat anti-mouse IgG (H + L) (A11029) from Life Technologies (Grand Island, NY, USA) were used for confocal microscopy. The secondary antibodies used for western blotting—DyLight 800 goat anti-mouse IgG (H + L) (RS23910) and DyLight 680 goat anti-rabbit IgG (H + L) (RS23720)—were obtained from Immunoway (Plano, TX, USA).

### 2.4. Transfection and Virus Titration

A549 cells were allowed to grow to approximately 70–80% confluency in 12-well plates and then transfected with the indicated plasmids for protein expression by using Lipofectamine 2000 reagent (Invitrogen, Carlsbad, CA, USA). Si_*KRT6A* or scrambled siRNA at a final concentration of 30 nM was transfected into approximately 5 × 10⁵ A549 cells seeded in 12-well plates by using the Lipofectamine RNAiMAX transfection reagent (Invitrogen). The siRNA knockdown efficiency of *KRT6A* was confirmed by means of RT-qPCR. Plasmid- or siRNA-transfected A549 cells grown in 12-well plates were infected with WSN (H1N1) virus at an MOI of 0.1. After virus adsorption for 1 h at 37 °C, the cells were washed with phosphate-buffered saline (PBS) and then incubated in F12K medium containing 0.125 μg/mL L-1-tosylamide-2-phenylmethyl chloromethyl ketone (TPCK)-treated trypsin (Worthington, Lakewood, NJ, USA) at 37 °C. Supernatants were collected at 24 and 48 h post-infection (p.i.), and virus titers were determined by means of plaque assays on MDCK cells, as described previously [43].

### 2.5. Co-Immunoprecipitation Assay

HEK293T cells were grown to approximately 70–80% confluency in 6-well plates and then transfected with the indicated plasmids by using the Lipofectamine LTX and Plus Reagents (Invitrogen). At 48 h post-transfection, the cells were lysed with IP lysis buffer (Pierce, Rockford, IL, USA) containing protease inhibitor cocktail (Roche, GmbH, Mannheim, Germany) on ice for 30 min and then centrifuged at 12,000 rpm at 4 °C for 10 min. Supernatants were incubated with the corresponding primary antibodies at 4 °C overnight, followed by the addition of protein A/G agarose (Roche) and incubation at 4 °C with rotation. After 6 h, the beads were washed three times with cold PBS. The immunoprecipitated proteins were then separated by SDS-PAGE and transferred onto nitrocellulose for western blotting.

### 2.6. Indirect Immunofluorescence Assay

A549 or HeLa cells grown on glass-bottom dishes were transfected with plasmids or siRNA, or further infected with WSN (H1N1) virus as indicated. The cells were fixed with 4% paraformaldehyde (PFA) for 30 min, permeabilized with 0.2% Triton X-100 for 30 min, and blocked with 1% BSA for 1 h at room temperature (RT). Incubation with the corresponding primary antibodies was carried out at 4 °C overnight, followed by further incubation with secondary antibodies conjugated to Alexa Fluor 488 or Alexa Fluor 633 for 1 h at RT. After three washes, the cells were incubated with DAPI (4’,6-diamidino-2-phenylindole; Thermo Fisher Scientific, Waltham, MA, USA) for 15 min at RT to stain the nuclei. Images were visualized using an LSM 980 with AiryScan confocal microscope (Zeiss, Oberkochen, Germany).

### 2.7. RNA Quantification

Total RNA of A549 cells was extracted by using an RNAsimple Total RNA Kit (Tiangen, Beijing, China) and was used to synthesize first-strand cDNA with a cDNA synthesis kit (Vazyme, Nanjing, China). Real-time PCR was performed by using ChamQ SYBR qPCR Master Mix (Vazyme). Relative RNA quantities were determined by using the ΔΔCt method and were normalized to the expression of the cellular GAPDH gene.

### 2.8. Nuclear and Cytoplasmic Fractionation

NE-PER Nuclear and Cytoplasmic Extraction Reagents (Thermo Fisher Scientific) were used for the separation of cytoplasmic and nuclear extracts. Briefly, the cell pellet was incubated with ice-cold CER I on ice for 10 min, followed by the addition of ice-cold CER II. After centrifugation at 12,000 rpm for 10 min at 4 °C, the supernatants were collected and used as cytoplasmic proteins. The insoluble fraction was suspended in ice-cold NER and vortexed at the highest setting for 15 s. The sample was then placed on ice and vortexed for 15 s every 10 min, for a total of 40 min. The extractions were centrifuged at 12,000 rpm for 15 min at 4 °C, and the supernatants were collected as the nuclear proteins.

### 2.9. Western Blotting

Protein samples were separated by SDS-PAGE using 10% or 12.5% polyacrylamide gel (EpiZyme), with 20 µL of protein extract loaded per well. Subsequently, the fractionated proteins were transferred onto nitrocellulose membranes (GE Healthcare) for Western blotting analysis. After being blocked with 5% skim milk in PBS, the membranes were incubated with primary antibodies diluted in PBS containing 0.5% BSA overnight at 4 °C and then washed three times with PBST. Following incubation with DyLight 800 goat anti-mouse IgG (H + L) or DyLight 680 goat anti-rabbit IgG (H + L) at RT for 1 h, the membranes were visualized using an Odyssey CLX infrared imaging system (LI-COR Biosciences).

### 2.10. Dual-Luciferase Reporter Assay

HEK293T cells were transfected with either si_*KRT6A* or scrambled siRNA. At 24 h post-transfection, the cells were further transfected with the plasmids of the minireplicon system, including the four RNP complex protein expression plasmids from the WSN (H1N1) virus (pCAGGS-PB2, pCAGGS-PB1, pCAGGS-PA, and pCAGGS-NP; 0.5 μg of each), the construct pHH21-SC09NS F-Luc (0.1 μg), and an internal control pRL-TK (0.1 μg). In a separate experiment, HEK293T cells were directly transfected with plasmids of the minireplicon system, together with a gradually increasing amount of Myc-KRT6A-expressing plasmids. Forty-eight hours later, cell lysates were prepared by using a dual-luciferase reporter assay system (Promega, Madison, WI, USA), with luciferase activities measured on a GloMax 96 microplate luminometer (Promega).

### 2.11. Cell Viability Assay

Cell viability was assessed by using a CellTiter-Glo luminescent cell viability assay, as described previously [43]. Briefly, A549 cells were treated with the indicated siRNA for 48 h. A total of 100 μL of CellTiter-Glo reagent (Promega) was then added directly into each well for 10 min at RT to lyse the cells. The luminescence of the cell lysates was measured using a GloMax 96 microplate luminometer.

### 2.12. Statistical Analysis

Data were statistically analyzed using a two-tailed unpaired Student’s t-test with GraphPad Prism 7.0 software (San Diego, CA, USA). A *p* value of <0.05 was considered to be statistically significant.

## 3. Results

### 3.1. KRT6A Negatively Regulates the Replication of IAV

**Figure 1:**
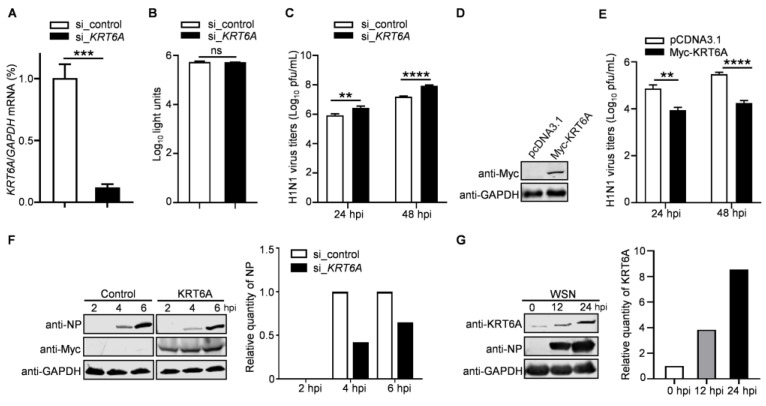
KRT6A negatively regulates the replication of IAV. (**A**) A549 cells were transfected with *KRT6A* siRNA (si_*KRT6A*) or with scrambled siRNA (si_control) for 48 h. Whole cell lysates were collected and analyzed by RT-qPCR. ***, *p* < 0.001. (**B**) Cell viability of si_*KRT6A*- or si_control-treated A549 cells as measured by using a CellTiter-Glo assay. ns, not significant. (**C**) A549 cells treated with si_*KRT6A* or si_control were infected with WSN (H1N1) virus at an MOI of 0.1. Supernatants were collected at 24 and 48 h p.i. and titrated for infectious viruses by means of plaque assay on MDCK cells. **, *p* < 0.01; ****, *p* < 0.0001. (**D**) A549 cells were transfected with plasmids expressing Myc-KRT6A or pCDNA3.1 vector, and the overexpression of Myc-KRT6A was confirmed by western blotting with a mouse anti-Myc mAb. (**E**) The Myc-KRT6A-overexpressing or control A549 cells were infected with WSN (H1N1) virus at an MOI of 0.1. Supernatants were collected at 24 and 48 h p.i. and titrated for infectious viruses by means of plaque assay on MDCK cells. **, *p* < 0.01; ****, *p* < 0.0001. (**F**) A549 cells were transfected with plasmids expressing Myc-KRT6A or pCDNA3.1 vector. At 48 h post-transfection, the cells were infected with WSN (H1N1) virus at an MOI of 5, and cell lysates were western blotted with a mouse anti-NP mAb at the indicated time points. (**G**) A549 cells were infected with WSN (H1N1) virus at an MOI of 0.1. Cell lysates were collected at 0, 12, and 24 h p.i. and western blotted with a mouse anti-KRT6A mAb.

KRT6A was identified as a potential regulator for the replication of IAV in a preliminary mass spectrometry screen. To confirm this finding, we analyzed the impact of siRNA-mediated *KRT6A* knockdown on the growth of IAV. Quantitative reverse transcription PCR (RT-qPCR) analysis showed that the level of *KRT6A* mRNA was reduced in A549 cells treated with *KRT6A*-specific siRNA (si_*KRT6A*) but not in scrambled siRNA (si_control)-treated cells (Figure 1A). Meanwhile, si_*KRT6A* treatment had no cytotoxic effects on A549 cells, as measured using a luminescent cell viability assay (Figure 1B). The siRNA-treated cells were infected with A/WSN/1933 (WSN, H1N1) virus (MOI = 0.1), and the viral growth titers in the supernatants were determined by plaque assay on MDCK cells. We found that *KRT6A* downregulation by siRNA treatment resulted in 3.3-/5.6-fold increases in virus titers in A549 cells at 24 and 48 h p.i. (Figure 1C), indicating that KRT6A plays a role in restricting the efficient replication of IAV.

We next evaluated the role of KRT6A in the replication of IAV in KRT6A-overexpressing or control cells. A549 cells were transfected with plasmids expressing Myc-KRT6A or empty pCDNA3.1 vector for 48 h (Figure 1D), followed by infection with WSN (H1N1) virus (MOI = 0.1). The culture supernatants were subjected to virus titration by plaque assay. We found that virus titers in KRT6A-overexpressing A549 cells were reduced by 11.1-/16.9-fold compared with those of control cells at 24 and 48 h p.i. (Figure 1E). These data further demonstrate that KRT6A negatively regulates IAV replication.

To corroborate the above findings, the KRT6A-overexpressing or control A549 cells were infected with WSN (H1N1) virus at an MOI of 5, and the expression of viral NP protein was detected by western blotting at 2, 4, and 6 h p.i. We found that viral NP levels in cells overexpressing KRT6A were remarkably reduced compared with those in cells transfected with empty vector (Figure 1F), indicating KRT6A may impair the early stage of the IAV replication cycle.

To further determine the biological importance of KRT6A in the replication of IAV, we also examined the expression level of KRT6A in response to IAV infection. A549 cells were infected with WSN (H1N1) virus at an MOI of 0.1, and the levels of KRT6A were determined by western blotting at 0, 12, and 24 h p.i. We found that along with the infection of IAV, the expression of KRT6A was gradually induced (Figure 1G). These data indicate that KRT6A plays a biological role in the course of IAV infection.

### 3.2. KRT6A Suppresses the Nuclear Import of Incoming vRNP Complex and Newly Synthesized NP

**Figure 2:**
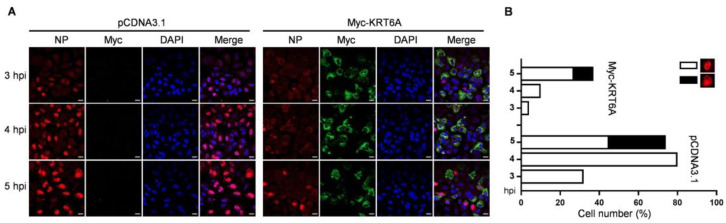
KRT6A inhibits the early stage of the IAV replication cycle. (**A**) A549 cells were transiently transfected with pCDNA3.1 vector or Myc-KRT6A-expressing plasmids. At 48 h post-transfection, the cells were infected with WSN (H1N1) virus at an MOI of 5. At 3, 4, and 5 h p.i., the cells were fixed and stained with a mouse anti-NP mAb and a rabbit anti-Myc pAb, followed by incubation with Alexa Fluor 488 goat anti-rabbit IgG (H + L) (green) and Alexa Fluor 633 goat anti-mouse IgG (H + L) (red). The nuclei were stained with DAPI (blue). Scale bar, 20 μm. (**B**) Statistical analysis of NP localization in virus-infected A549 cells as indicated in (**A**). The localization of NP after nuclear import was classified into two types: clear nuclear localization and predominant nuclear localization. The results are counted from one hundred cells observed under a confocal microscope with a 60× objective lens.

To investigate the stage of the IAV replication cycle in which KRT6A functions, A549 cells were transfected with plasmids expressing Myc-KRT6A or empty pCDNA3.1 vector. At 48 h post-transfection, the cells were infected with WSN (H1N1) virus at an MOI of 5. The cellular localization of viral NP (indicative of the localization of the vRNP complex) and KRT6A was visualized by means of confocal microscopy at 3, 4, and 5 h p.i. (Figure 2A,B). We found that KRT6A was predominantly localized in the cytoplasm of Myc-KRT6A-overexpressing cells across the three time points p.i. Notably, viral NP was clearly accumulated in the nucleus of 32%, 80%, and 45% of control cells at 3, 4, and 5 h p.i., respectively. By contrast, only 4%, 10%, and 27% of KRT6A-overexpressing cells exhibited visible nuclear accumulation of NP at the same time points. These results indicate that the overexpression of Myc-KRT6A significantly inhibits the early stage of the IAV replication cycle.

**Figure 3:**
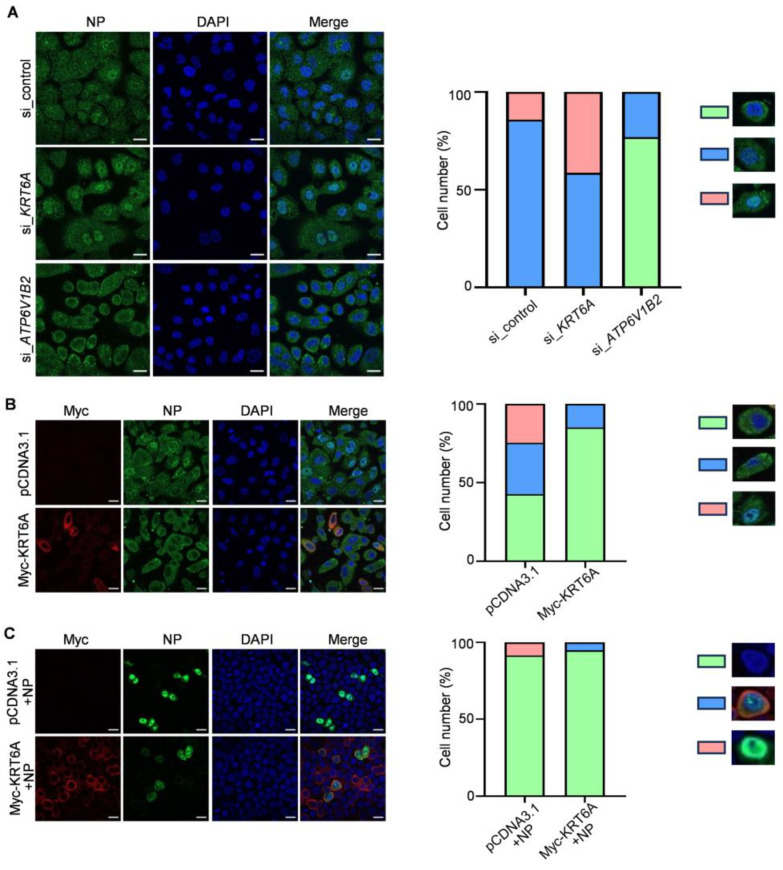
KRT6A inhibits the nuclear import of the incoming vRNP complex and newly synthesized NP. (**A**) A549 cells were transfected with si_control, si_*KRT6A*, or si_*ATP6V1B2*. At 48 h post-transfection, the cells were incubated with 50 μg/mL CHX for 1 h to inhibit protein synthesis. The treated cells were infected with WSN (H1N1) virus at an MOI of 50 on ice, followed by incubation in a culture medium containing CHX for another 4 h at 37 °C. The cells were then fixed, incubated with a mouse anti-NP mAb, and stained with Alexa Fluor 488 goat anti-mouse IgG (H + L). (**B**) A549 cells were transfected with plasmids expressing Myc-KRT6A or pCDNA3.1 vector. At 48 h post-transfection, the cells were treated with 50 μg/mL CHX for 1 h. The treated cells were infected with WSN (H1N1) virus at an MOI of 50 on ice, followed by incubation in a culture medium containing CHX for another 5 h at 37 °C. The cells were then fixed, incubated with a mouse anti-NP mAb and a rabbit anti-Myc pAb, and stained with Alexa Fluor 488 goat anti-mouse IgG (H + L) and Alexa Fluor 633 goat anti-rabbit IgG (H + L). (**C**) HeLa cells grown on glass bottom dishes were co-transfected with plasmids expressing WSNNP and Myc-KRT6A or pcDNA3.1 vector. At 48 h post-transfection, the cells were fixed, incubated with a mouse anti-NP mAb and a rabbit anti-Myc pAb, and stained with Alexa Fluor 488 goat anti-mouse IgG (H + L) and Alexa Fluor 633 goat anti-rabbit IgG (H + L). The nuclei were stained with DAPI (**A**–**C**). Scale bar, 20 μm.

The nuclear import of the vRNP complex was mediated by the interaction of NP with the classical nuclear import pathway. We then investigated whether KRT6A regulates the nuclear import of vRNP during IAV infection. To this end, A549 cells were treated with si_control, si_*KRT6A,* or siRNA targeting vATPase subunit ATP6V1B2 gene (si_*ATP6V1B2*) for 48 h, followed by infection with WSN (H1N1) virus at an MOI of 50 in the presence of cycloheximide (CHX) to inhibit the synthesis of new proteins. At 4 h p.i., the nuclear import of the incoming vRNP complex was visualized by confocal microscopy. We found that following si_control treatment, viral NP was simultaneously detected in the nucleus and cytoplasm in 85.8% of cells, and was clearly accumulated in the nucleus in 14.2% of cells, indicating that the nuclear import of the incoming vRNP complex had occurred. By contrast, due to the inhibition of the viral uncoating step caused by ATP6V1B2 downregulation, the vRNP complex was predominantly retained in the cytoplasm of si_*ATP6V1B2*-treated cells. However, 41.4% of si_*KRT6A*-treated cells showed apparent nuclear accumulation of viral NP, which was much higher than that of the si_control-treated cells (Figure 3A). Since the only source of NP protein was derived from the incoming vRNP complex under the treatment of CHX, these results demonstrate that KRT6A directly inhibits the nuclear import of the incoming vRNP complex during the early stage of the virus replication cycle.

We also examined the effect of KRT6A overexpression on the nuclear import of the incoming vRNP complex. A549 cells were transfected with plasmids expressing Myc-KRT6A or pCDNA3.1 vector. At 48 h post-transfection, the cells were infected with WSN (H1N1) virus at an MOI of 50 in the presence of CHX treatment. At 5 h p.i., the nuclear import of the incoming vRNP complex was visualized by confocal microscopy. We found that viral NP was accumulated in the nucleus in 24.6% of control cells; in contrast, viral NP was predominantly distributed in the cytoplasm of Myc-KRT6A-overexpressing cells (Figure 3B). These data confirmed that KRT6A functions to inhibit the nuclear import of the incoming vRNP complex.

We further determined the effect of KRT6A on the cellular localization of viral NP when they were co-expressed. To this end, Hela cells were used for confocal microscopy to exclude the possibility of cell type-specific effect. The cells were transfected with plasmids expressing viral NP and Myc-KRT6A individually or in combination, and the distribution of NP and KRT6A was examined by confocal microscopy at 48 h post-transfection. We found that NP was dominantly accumulated in the nucleus of the control cells, whereas its nuclear accumulation was dramatically inhibited when KRT6A was co-expressed (Figure 3C). These data, together with the above results, demonstrate that KRT6A inhibits the nuclear import of the incoming vRNP complexes and newly synthesized NP, thereby suppressing the virus replication cycle of IAV.

### 3.3. KRT6A Interacts with RNP Complex Proteins of IAV

**Figure 4:**
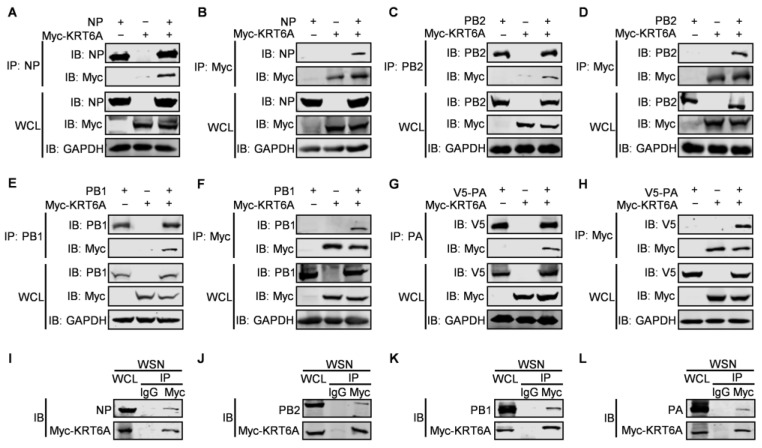
KRT6A interacts with RNP complex proteins. (A-H) HEK293T cells were transfected individually or in combination with plasmids expressing Myc-KRT6A and WSNNP (**A**,**B**), WSNPB2 (**C**,**D**), WSNPB1 (**E**,**F**), or V5-WSNPA (**G**,**H**). Cell lysates were immunoprecipitated with a mouse anti-NP mAb (**A**), anti-PB2 mAb (**C**), anti-PB1 mAb (**E**), anti-PA mAb (**G**), or a mouse anti-Myc mAb (**B**,**D**,**F**,**H**). The immunoprecipitated proteins were western blotted with a rabbit anti-Myc pAb and a rabbit anti-NP pAb (**A**,**B**), anti-PB2 pAb (**C**,**D**), anti-PB1 pAb (**E**,**F**), or anti-V5 pAb (**G**,**H**). (**I**–**L**) HEK293T cells were transfected with Myc-KRT6A-expressing plasmids, and at 48 h post-transfection, the cells were infected with WSN (H1N1) virus at an MOI of 5. At 8 h p.i., cell lysates were immunoprecipitated with a mouse IgG or a mouse anti-Myc mAb, and the immunoprecipitated proteins were western blotted with a rabbit anti-Myc pAb and a rabbit anti-NP pAb (**I**), anti-PB2 pAb (**J**), anti-PB1 pAb (**K**), or anti-PA pAb (**L**).

To determine whether KRT6A interacts with the component proteins of the RNP complex of IAV, HEK293T cells were transfected with plasmids expressing Myc-tagged KRT6A and NP, PB2, PB1, or V5-PA of WSN (H1N1) virus, either alone or in combination. At 48 h post-transfection, cell lysates were subject to immunoprecipitation with a mouse anti-NP, PB2, PB1, or PA monoclonal antibody (mAb), followed by western blotting with a rabbit anti-Myc polyclonal antibody (pAb) and a rabbit anti-NP, anti-PB2, anti-PB1, or anti-V5 pAb. We found that Myc-KRT6A was co-immunoprecipitated with WSN NP, PB2, PB1, or PA when they were co-expressed (Figure 4A,C,E,G). Meanwhile, WSN NP, PB2, PB1, or PA was also co-immunoprecipitated with Myc-KRT6A when the co-IP experiment was performed with a mouse anti-Myc mAb (Figure 4B,D,F,H). Together, these results indicate that KRT6A interacts with IAV NP, PB2, PB1, and PA in transiently transfected cells.

To further investigate the interaction between RNP complex proteins and KRT6A during IAV infection, HEK293T cells were transfected to express Myc-KRT6A. At 48 h post-transfection, the cells were infected with WSN (H1N1) virus at an MOI of 5. After 8 h, the cell lysates were immunoprecipitated with a mouse anti-Myc mAb or mouse IgG, and the presence of RNP complex proteins and KRT6A in the immunoprecipitates was revealed by western blotting. We found that the four RNP complex proteins interacted with KRT6A in the course of IAV infection (Figure 4I–L).

### 3.4. KRT6A Has No Effect on the Nuclear Import of PB2 or PB1-PA Heterodimer

**Figure 5:**
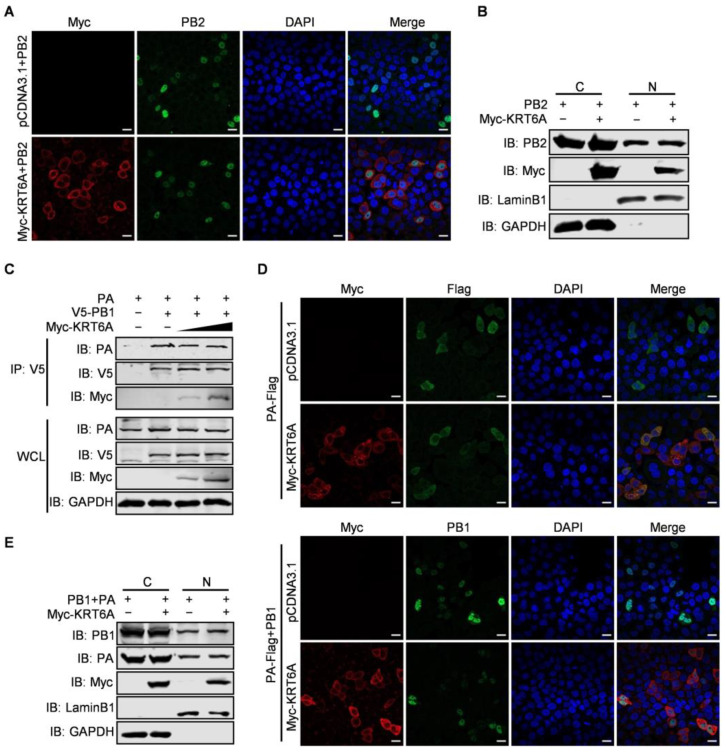
KRT6A does not affect the nuclear import of PB2 or the PB1-PA heterodimer. (**A**) HeLa cells grown on glass bottom dishes were co-transfected with plasmids expressing WSNPB2 and Myc-KRT6A or pcDNA3.1 vector. At 48 h post-transfection, the cells were fixed, incubated with a mouse anti-PB2 mAb and a rabbit anti-Myc pAb, and stained with Alexa Fluor 488 goat anti-mouse IgG (H + L) and Alexa Fluor 633 goat anti-rabbit IgG (H + L). DAPI was used to stain the nuclei. (**B**) HEK293T cells were co-transfected with plasmids expressing WSNPB2 and Myc-KRT6A or pCDNA3.1 vector. Forty-eight hours later, the cells were separated into nuclear (N) and cytoplasmic (C) fractions. Each fraction was western blotted with a mouse anti-PB2 mAb and a rabbit anti-Myc pAb for protein detection. LaminB1 and GAPDH were used as nuclear and cytoplasmic marker proteins, respectively. (**C**) HEK293T cells were transfected with the indicated combinations of plasmids. At 48 h post-transfection, the cell lysates were immunoprecipitated with a mouse anti-V5 mAb, and the immunoprecipitated proteins were western blotted with a rabbit anti-PA pAb, anti-V5 pAb, and anti-Myc pAb. (**D**) HeLa cells grown on glass bottom dishes were transfected with the indicated combination of plasmids to express WSNPA-Flag together with or without WSNPB1, Myc-KRT6A, and empty pCDNA3.1 vector. At 48 h post-transfection, the cells were incubated with a rabbit anti-Myc pAb and a mouse anti-Flag mAb or a mouse anti-PB1 mAb, and stained with Alexa Fluor 633 goat anti-rabbit IgG (H + L) and Alexa Fluor 488 goat anti-mouse IgG (H + L). DAPI was used to stain the nuclei. (E) HEK293T cells were transfected with plasmids expressing WSNPB1, WSNPA, and Myc-KRT6A or pCDNA3.1 vector. Forty-eight hours later, the cells were separated into nuclear (N) and cytoplasmic (C) fractions. Each fraction was western blotted with a mouse anti-PB1 mAb, a mouse anti-PA mAb, and a rabbit anti-Myc pAb for protein detection.

Given that KRT6A interacts with all four component proteins of the vRNP complex and suppresses the nuclear import of NP protein as well as the vRNP complex as a whole, we then asked whether KRT6A also affects the nuclear import of PB2, PB1, and PA protein. At first, we examined the effect of KRT6A on the nuclear import of the viral PB2 protein. HeLa cells were transfected with plasmids expressing WSN PB2 together with Myc-KRT6A-expressing plasmids or pCDNA3.1 vector and were subjected to confocal microscopy at 48 h post-transfection. We found that PB2 was similarly transported into the nucleus of both control cells and KRT6A-overexpressing cells (Figure 5A). To confirm this finding, we performed a nuclear and cytoplasmic fractionation assay in HEK293T cells that were co-transfected with plasmids expressing WSN PB2 and Myc-KRT6A or pCDNA3.1 vector. At 48 h post-transfection, nuclear and cytoplasmic proteins were separated and analyzed by western blotting, with LaminB1 and GAPDH used as nuclear and cytoplasmic marker proteins, respectively. As shown in Figure 5B, KRT6A was primarily localized in the cytoplasm and was less abundantly detected in the nucleus of KRT6A-overexpressing cells. Notably, the expression of PB2 in the cytoplasm and nucleus was comparable between control cells and KRT6A-overexpressing cells. These data further demonstrate that the nuclear import of PB2 is not affected by KRT6A.

In the course of IAV infection, newly synthesized PB1 and PA proteins form heterodimers in the cytoplasm before they are imported into the nucleus of infected cells [44]. To examine the effect of KRT6A on the nuclear import of the PB1-PA heterodimer, we first determined whether it affects the interaction between PB1 and PA. HEK293T cells were transfected with plasmids expressing V5-PB1 and PA of WSN (H1N1) virus, together with or without Myc-KRT6A-expressing plasmids. At 48 h post-transfection, the cell lysates were immunoprecipitated with a mouse anti-V5 mAb and then subjected to western blotting with a rabbit anti-V5 pAb, anti-PA pAb, or anti-Myc pAb. We found that the amount of PA co-immunoprecipitated with PB1 was similar in the absence or presence of two different doses of KRT6A (Figure 5C), indicating that co-expression of KRT6A had no effect on the formation of the PB1-PA heterodimer. Next, we performed a confocal microscopy assay to monitor the nuclear import of the PB1-PA dimer in the absence or presence of KRT6A. HeLa cells were transfected with plasmids to express PA-Flag together with or without PB1, Myc-KRT6A, and empty pCDNA3.1 vector. We found that at 48 h post-transfection, PA-Flag was dominantly localized in the cytoplasm when it was expressed alone, and its cellular localization was not affected by the co-expression of Myc-KRT6A (Figure 5D). When the localization of PB1 was visualized to indicate the nuclear import of PB1-PA dimer, we found that PB1 was accumulated in the nucleus of cells co-expressing PB1 and PA-Flag. Notably, the nuclear accumulation of PB1 in the presence of PA-Flag was not affected by the co-expression of Myc-KRT6A (Figure 5D). Nuclear and cytoplasmic fractionation assay in HEK293T cells also showed no differences in the protein levels of nucleus-localized PB1 and PA between KRT6A-overexpressing and control cells (Figure 5E). Together, these results demonstrate that KRT6A does not affect the nuclear import of the PB1-PA heterodimer in the replication cycle of IAV.

### 3.5. KRT6A Weakens the Binding of NP with Importin α3

**Figure 6:**
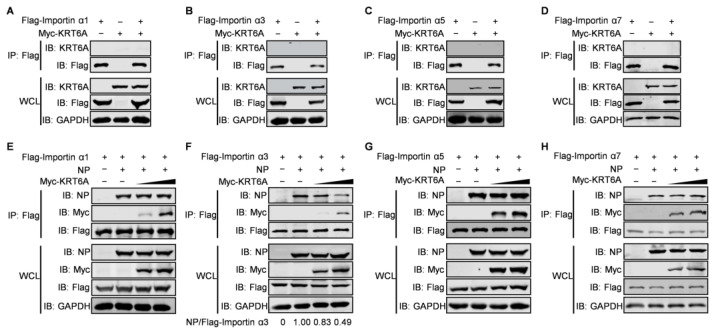
KRT6A weakens the binding of NP to importin α3. (**A**–**D**) HEK293T cells were transfected individually or in combination with plasmids expressing Myc-KRT6A and Flag-tagged importin α1, α3, α5, or α7. Cell lysates were immunoprecipitated with a mouse anti-Flag mAb, and the immunoprecipitated proteins were western blotted with a rabbit anti-KRT6A pAb and anti-Flag pAb. (**E**–**H**) HEK293T cells were transfected with plasmids expressing WSNNP and Flag-tagged importin α1, α3, α5, or α7, in the absence or presence of gradually increasing amounts of Myc-KRT6A. Cell lysates were immunoprecipitated with a mouse anti-Flag mAb, and the immunoprecipitated proteins were western blotted with a rabbit anti-NP pAb, anti-Flag pAb, and anti-Myc pAb.

The nuclear import of NP is mediated by its association with importin α1, α3, α5, or α7 and the subsequent binding of importin β1 [27,28,45]. Given that KRT6A interacts with NP and hinders the nuclear import of the vRNP complex and newly synthesized NP, we hypothesized that KRT6A might affect the interaction between NP and importin α isoforms. To test this hypothesis, we first determined whether KRT6A interacts with importin α isoforms by performing co-IP assays. HEK293T cells were transfected with plasmids expressing Myc-KRT6A and Flag-tagged importin α1, α3, α5, or α7, individually or in combination. Cell lysates were immunoprecipitated with a mouse anti-Flag mAb, and then subjected to western blotting with a rabbit anti-Flag or anti-KRT6A pAb. As shown in Figure 6A–D, KRT6A did not interact with importin α1, α3, α5, or α7.

We further examined whether KRT6A affects complex formation between NP and importin α isoforms. HEK293T cells were transfected with plasmids expressing WSN NP and Flag-tagged importin α1, α3, α5, or α7, together with gradually increasing amounts of Myc-KRT6A-expressing plasmids. At 48 h post-transfection, the cell lysates were immunoprecipitated with a mouse anti-Flag mAb and then subjected to western blotting with a rabbit anti-Flag, anti-Myc, or anti-NP pAb. We found that the amount of NP co-immunoprecipitated with Flag-tagged importin α1, α5, or α7 was not affected in the presence of gradually increasing amounts of Myc-KRT6A (Figure 6E,G,H). By contrast, the co-expression of gradually increasing amounts of Myc-KRT6A reduced the amount of NP co-immunoprecipitated with importin α3 in a dose-dependent manner (Figure 6F). These results demonstrate that the presence of KRT6A specifically interferes with the formation of a complex between NP and importin α3, thereby inhibiting the nuclear import of the incoming vRNP complex and newly synthesized NP.

### 3.6. KRT6A Impairs vRNP Complex Assembly

**Figure 7:**
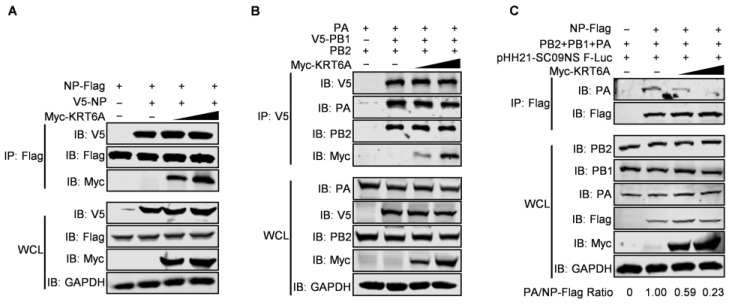
KRT6A impairs vRNP complex assembly. (**A**) HEK293T cells were transfected with plasmids expressing WSNNP-Flag and V5-WSNNP, along with increasing amounts of Myc-KRT6A-expressing plasmids. At 48 h post-transfection, cell lysates were immunoprecipitated with a mouse anti-Flag mAb, and the immunoprecipitated proteins were western blotted with a rabbit anti-Flag pAb, anti-V5 pAb, and anti-Myc pAb. (**B**) HEK293T cells were transfected with plasmids expressing WSNPB2, V5-WSNPB1, and WSNPA, along with increasing amounts of Myc-KRT6A-expressing plasmids. At 48 h post-transfection, cell lysates were immunoprecipitated with a mouse anti-V5 mAb, and the immunoprecipitated proteins were western blotted with a rabbit anti-PB2 pAb, anti-V5 pAb, anti-PA pAb, and anti-Myc pAb. (**C**) HEK293T cells were transfected with plasmids expressing WSNPB2, WSNPB1, WSNPA, WSNNP-Flag, and pHH21-SC09NS F-Luc, along with increasing amounts of Myc-KRT6A-expressing plasmids. At 48 h post-transfection, cell lysates were immunoprecipitated with a mouse anti-Flag mAb, and the immunoprecipitated proteins were western blotted with a rabbit anti-PA pAb and anti-Flag pAb.

IAV NP forms homo-oligomers and multiple copies of NP wrap around genomic RNA, which provides a structural framework for the assembly of the vRNP complex [19,46]. We therefore performed a co-IP assay to determine whether KRT6A affects the homo-oligomerization of NP. HEK293T cells were co-transfected with plasmids expressing V5-NP and NP-Flag of WSN (H1N1) virus, together with or without Myc-KRT6A-expressing plasmids. At 48 h post-transfection, cell lysates were immunoprecipitated with a mouse anti-Flag mAb and then subjected to western blotting with a rabbit anti-Flag, anti-V5, or anti-Myc pAb. We found that the co-expression of increasing amounts of Myc-KRT6A did not affect the interaction of V5-NP and NP-Flag (Figure 7A), indicating the lack of effect of KRT6A on the homo-oligomerization of NP.

The interaction between KRT6A and the three polymerase subunits of IAV also prompted us to examine whether it affects the formation of the integral viral polymerase complex. The co-IP assay performed in HEK293T cells showed that the amounts of PB2 and PA protein co-immunoprecipitated with V5-PB1 were not affected by the gradual increase of KRT6A expression (Figure 7B), thereby indicating that KRT6A has no effect on the formation of the polymerase complex of IAV.

Given that the transcription and replication of the viral genome is catalyzed by the vRNP complex of IAV, we further determined whether the interaction between KRT6A and all four component proteins of the vRNP complex affects the formation of the vRNP complex. To this end, we performed a co-IP assay in HEK293T cells transfected with plasmids expressing PB2, PB1, PA, NP-Flag, and virus-like RNA to constitute the vRNP complex, together with a gradually increasing amount of KRT6A-expressing plasmids. We found that the amount of PA protein co-immunoprecipitated with NP was dramatically reduced along with the gradual increase of KRT6A expression (Figure 7C). Because viral PA does not directly interact with NP and can only be co-immunoprecipitated by NP in the vRNP complex [47], these results indicate that the expression of KRT6A impairs the formation of functional vRNP complexes of IAV.

### 3.7. KRT6A Reduces the Polymerase Activity of IAV

**Figure 8:**
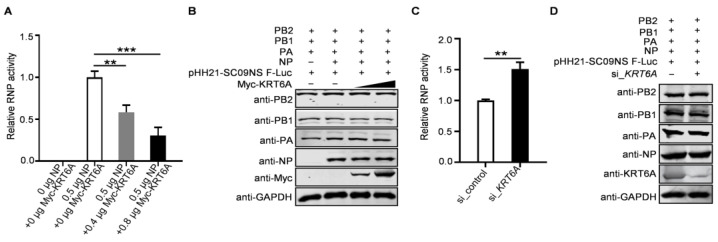
KRT6A reduces the polymerase activity of IAV. (**A**,**B**) HEK293T cells were transfected with plasmids expressing the four RNP complex proteins (PB2, PB1, PA, and NP) of WSN (H1N1) virus, pHH21-SC09NS-F-Luc, pRL-TK, and Myc-KRT6A-expressing plasmids. At 48 h post-transfection, a dual-luciferase assay was performed, and the relative firefly luciferase activity was normalized to the value of Renilla luciferase activity (**A**). **, *p* < 0.01; ***, *p* < 0.001. The cell lysates were also western blotted with a mouse anti-PB2 mAb, anti-PB1 mAb, anti-PA mAb, anti-NP mAb, and anti-Myc mAb to detect the corresponding proteins (**B**). (**C**,**D**) HEK293T cells were transfected with si_*KRT6A* or scrambled siRNA. At 24 h post-transfection, the siRNA-treated cells were further transfected with plasmids expressing the four RNP complex proteins (PB2, PB1, PA, and NP) of WSN (H1N1) virus, pHH21-SC09NS-F-Luc, and pRL-TK. At 48 h post-transfection, a dual-luciferase assay was performed, and the relative firefly luciferase activity was normalized to the value of Renilla luciferase activity (**C**). **, *p* < 0.01. The cell lysates were also western blotted with a mouse anti-PB2 mAb, anti-PB1 mAb, anti-PA mAb, anti-NP mAb, and anti-KRT6A pAb to detect the corresponding proteins (**D**).

As the formation of a functional vRNP complex is required for the viral polymerase to catalyze the transcription and replication of the viral genome, we finally explored the impact of KRT6A on viral polymerase activity by performing a minigenome assay. HEK293T cells were transfected with plasmids expressing the four RNP complex proteins of WSN (H1N1) virus, the construct pHH21-SC09NS F-Luc for the synthesis of virus-like RNA, and the internal control pRL-TK, together with increasing amounts of Myc-KRT6A-expressing plasmids. We found that viral polymerase activity was reduced due to the overexpression of KRT6A in a dose-dependent manner (Figure 8A). Meanwhile, KRT6A overexpression did not affect the expression levels of PB2, PB1, PA, and NP (Figure 8B), indicating that KRT6A inhibits viral polymerase activity without affecting the expression levels of RNP component proteins. Conversely, we found that siRNA-mediated KRT6A knockdown led to increased viral polymerase activity without affecting the expression levels of RNP complex proteins (Figure 8C,D). Together, these results indicate that KRT6A suppresses the viral polymerase activity of IAV.

## 4. Discussion

The replication cycle of IAV begins with binding to the sialic acid receptors on the surface of target cells [48]. After binding, the virus is internalized and delivered into early endosomes [49,50]. The pH decrease as the virus moves from the early to late endosome alters the conformation of the viral HA protein, resulting in the exposure of the fusion peptide and consequent fusion between the viral envelope and late endosomal membranes [51,52,53]. Further acidification of the interior of the virus particle finally uncoats the virion, resulting in the release of the vRNP complex into the cytoplasm [54,55]. vRNPs are subsequently imported into the nucleus of the infected cells via the classical nuclear import pathway, which is mediated by the interaction between the NLSs of NP and isoforms of importin α, i.e., α1, α3, α5, and α7 [27,28,45]. Upon entering the nucleus, the incoming vRNPs begin a primary round of transcription, producing mRNA from which progeny viral proteins, including PB2, PB1, PA, and NP, are translated [47]. PB1 and PA form heterodimers in the cytoplasm, which are transported to the nucleus with the aid of the nuclear import receptor Ran-binding protein 5 (RanBP5) [56]. By contrast, the NLSs of PB2 and NP bind isoforms of importin α to access the classical nuclear import pathway [17,57,58,59,60]. In the nucleus, the newly synthesized polymerase and NP proteins interact with nascent viral RNA to assemble into progeny vRNP complexes.

A couple of host cellular proteins are capable of promoting the nuclear import of vRNP complexes and/or newly synthesized component proteins through different mechanisms. For example, BinCARD1 can specifically enhance the binding between NP and importin α7 [22]; and Hsp40 binds to NP through its J domain and recruits importin α isoforms through its C-terminus, thereby facilitating NP–importin α interaction [61]. On the other hand, several other host factors can inhibit the nuclear import of the vRNP complex and/or its component proteins. Among them, PLSCR1 forms a trimeric complex with NP and importin α, thereby blocking the further recruitment of importin β [27]; eEF1D weakens the interactions of NP and importin α5 as well as PB1 and RanBP5 to impede the nuclear import of NP and the PB1-PA heterodimer [62]; MOV10 interacts with NP to prevent the further binding of NP with importin α [28]; and HCLS1-associated protein X1 (HAX1) interacts with the NLS domain of PA and impedes its nuclear import [63]. In the present study, we identified KRT6A as a negative regulator of the replication of IAV. siRNA-mediated knockdown of *KRT6A* mRNA level increased the replication of IAV, whereas overexpression of KRT6A reduced virus growth titers. We demonstrated that KRT6A interacted with viral PB2, PB1, PA, and NP proteins in both transfected and infected cells. Of note, KRT6A affected the nuclear import of incoming vRNP complexes and newly synthesized NP but had no effect on the nuclear import of the PB1-PA heterodimer or PB2 protein. Given that the vRNP complexes of IAV utilize the NLSs of NP for their nuclear import, we further explored the mechanism by which KRT6A affected the nuclear import of the vRNP complex and NP. We found that, among the different isoforms of importin α that interact with NP, KRT6A specifically interfered with the binding between importin α3 and NP. Further investigation also demonstrated that KRT6A impaired the assembly of the vRNP complex and reduced viral polymerase activity. Together, these negative effects of KRT6A on nuclear import and function of the vRNP complex contributed to suppressing the propagation of IAV.

Being one of the critical steps of the IAV replication cycle, the nuclear import of vRNP complexes becomes the key node where proviral and antiviral host factors function. Interestingly, some host factors such as Hsp40 and MOV10 indiscriminately affect the interaction between NP and different isoforms of importin α, whereas other host factors such as BinCARD1, eEF1D, and KRT6A differentially affect the interaction of NP with certain isoforms of importin α. Although different importin α members share general structural features, their sequence homology can be as low as 41% [64]. The finding that some host factors specifically affect the interaction between NP and certain importin α members may reflect the subtle difference of the importin α family members in their interaction with viral NP protein. Since KRT6A does not interact with importin α3, it may directly compete with importin α3 for the binding with NP, thereby inhibiting the nuclear import of vRNP complexes and NP.

At present, the role of keratins in IAV replication has been gradually uncovered. An influenza virus–host interactome screen by Watanabe et al. indicated that KRT14 plays an important role in early steps of the IAV life cycle, such as virus binding, internalization, and/or transport of vRNP complexes into the nucleus [65]. The replication of IAV has been shown to induce KRT8 phosphorylation, which enhances viral replication efficiency in A549 cells [66]. In addition, proteomic analyses in H9N2 avian influenza virus-infected A549 cells demonstrated that some keratins (i.e., KRT1, KRT10, KRT14, KRT16, KRT18, and KRT19) were differentially expressed [67,68], indicating that these cytoskeleton molecules may be involved in the replication cycle of IAV. In this study, we identified KRT6A as a restriction factor for the replication of IAV and elucidated its underlying functional mechanism. Our study thus clearly demonstrates the role of keratins in the replication of IAV.

In summary, our data demonstrate that KRT6A is a novel NP-binding protein that has a negative regulatory effect on IAV replication. KRT6A suppresses the nuclear import of the vRNP complex and newly synthesized NP, thereby impeding the replication cycle of IAV. Notably, we unveiled the underlying mechanism by which KRT6A functions, namely by impairing the formation of a complex between viral NP and the nuclear import adaptor importin α3. Furthermore, KRT6A also inhibits the assembly of functional vRNP complexes and consequently reduces viral polymerase activity. Our findings thus comprehensively reveal the role of KRT6A in the replication cycle of IAV, which advances our understanding of the interaction network between IAV and host cellular factors.

## 5. Conclusions

In summary, our results demonstrated that the KRT6A–NP interaction impairs the nuclear import of incoming vRNP complexes and newly synthesized NP. KRT6A specifically interferes with the interaction between NP and importin α3. Consequently, the inhibitory effect of KRT6A on the nuclear import of NP suppresses the assembly of vRNP complexes and viral polymerase activity.

## Data Availability

The data presented in this study are included in the article. Further inquiries can be directed to the corresponding authors.
